# Data on microRNA expression, predicted gene targets and pathway analysis in response to different concentrations of a cranberry proanthocyanidin-rich extract and its metabolite 3-(4-hydroxyphenyl)-propionic acid in intestinal Caco-2BBe1 cells

**DOI:** 10.1016/j.dib.2024.110238

**Published:** 2024-03-06

**Authors:** Zoe Dimoff, Zoe Lofft, Fred Liang, Siying Chen, Paraskevi Massara, Diana Wu, Inke Paetau-Robinson, Christina Khoo, Amel Taibi, Elena M. Comelli

**Affiliations:** a Department of Nutritional Sciences^2^, University of Toronto, ON, Canada; b Ocean Spray Cranberries, Inc., Lakeville-Middleboro, MA 02349, USA; c Joannah and Brian Lawson Centre for Child Nutrition, University of Toronto, ON, Canada

**Keywords:** Cranberry proanthocyanidin, 3-(4-hydroxyphenyl)-propionic acid, microRNA, Intestine, Caco-2BBe1 cells

## Abstract

Cranberry-derived proanthocyanidin (PAC) is processed by the gut microbiota to produce 3-(4-hydroxyphenyl)-propionic acid (HPPA), among other metabolites. These data are in support of the article entitled, “Cranberry proanthocyanidin and its microbial metabolite 3,4-dihydroxyphenylacetic acid, but not 3-(4-hydroxyphenyl)-propionic acid, partially reverse pro-inflammatory microRNA responses in human intestinal epithelial cells,” published in Molecular Nutrition and Food Research [1]. Here we describe data generated by nCounter^Ⓡ^ Human v3 miRNA Expression Panel of RNA obtained from Caco-2BBe1 cells exposed to two different concentrations of cranberry extract rich in PAC (50 µg/ml or 100 µg/ml) or 3-(4-hydroxyphenyl)-propionic acid (5 µg/ml or 10 µg/ml) for 24 h, then stimulated with 1 ng/ml of IL-1ß or not (mock) for three hours. The raw data are publicly available at the NCBI GEO database GSE237078. This work also includes descriptive methodological procedures, treatment-responsive microRNA (miRNA) expression profiles in Caco-2BBe1 cells, and *in silico* mRNA gene target and pathway enrichment analyses of significantly differentially expressed miRNAs (*q* < 0.001). Cranberry and its components have recognized health benefits, particularly in relation to combatting inflammation and pathogenic bacterial adhesion. These data will be valuable as a reference to study the response of intestinal cells to other polyphenol-rich food sources, analyze gut microbial responses to cranberry and its metabolites in different cell lines and mammalian hosts to elucidate individualized effects, and to delineate the role of the gut microbiota in facilitating the benefits of cranberry. Moreover, these data will aid in expanding our knowledge on the mechanisms underlying the benefits of cranberry and its components.

Specifications Table

**Table:** 

Subject	Genetics, Epigenetics
Specific subject area	Nutrition, MicroRNA, intestinal homeostasis
Type of data	Table Figure Reporter Code Count (RCC) files corresponding to the raw miRNA data collected using the Nanostring digital analyzer Txt files extracted from the RCC files using the Nanostring nSolver software.
How data were acquired	Expression profiling of 799 miRNA using nCounter^Ⓡ^ Human v3 miRNA Expression Panel (Nanostring Technologies, Seattle, WA, USA; miRBase built version 21 at Princess Margaret Genomic Centre, Toronto, ON, Canada.
Data format	Raw Analyzed Filtered
Description of data collection	Total RNA was extracted from the cell pellets from the 10 treatment groups. MicroRNA profiling was performed using the nCounter^Ⓡ^ Human v3 miRNA Expression Panel. Data processing was performed using the R package NanoStringNorm and statistical analyses were performed using the R package NanoStringDiff. *In silico* gene target predictions were determined using mirDIP and pathway enrichment analyses were performed using pathDIP.
Data source location	Institution: University of Toronto City/Town/Region: Toronto/Ontario Country: Canada Latitude and longitude (and GPS coordinates, if possible) for collected samples/data: 43.6629° N, 79.3957° W
Data accessibility	Both the raw and processed miRNA data were deposited in a public repository under the accession number GSE237078. Repository name: National Center for Biotechnology Information (NCBI) Gene Expression Omnibus (GEO) database Data identification number: GSE237078 Direct URL to data: https://www.ncbi.nlm.nih.gov/geo/query/acc.cgi?acc=GSE237078
Related research article	Lofft Z, Taibi A, Massara P, Tokar T, Paetau‐Robinson I, Khoo C, et al. Cranberry Proanthocyanidin and Its Microbial Metabolite 3,4‐Dihydroxyphenylacetic Acid, but Not 3‐(4‐Hydroxyphenyl)‐Propionic Acid, Partially Reverse Pro‐Inflammatory microRNA Responses in Human Intestinal Epithelial Cells. Molecular Nutrition Food Res 2022;66:2100853. 10.1002/mnfr.202100853.

## 1 Value of the Data

• These data provide the first profile of the intestinal miRNA response to different doses of a cranberry proanthocyanidin-enriched extract and a proanthocyanidin gut microbial-derived metabolite, with and without inflammation. This is important because mechanisms underlying effects of cranberry in the intestinal tract are incompletely understood. Understanding miRNA variation may help to decipher host responses in health and disease.
• These data will provide an important reference for scientists studying miRNA-modulatory effects of polyphenol-rich foods or different dietary sources of proanthocyanidin, and to conduct dose-response studies. The data may also help scientists and clinical practitioners in developing new approaches targeting miRNAs to sustain intestinal health.
• These data can be used in multi-omics analyses examining the effects of cranberry PAC and its metabolites on the intestine and to identify miRNA biomarkers that may predict the utility of cranberry-based dietary interventions aimed at maintaining intestinal homeostasis or countering inflammation.

## 2 Data Description

This manuscript provides miRNA expression data obtained from intestinal epithelial Caco-2BBe1 cells exposed to cranberry-proanthocyanidin (PAC)-rich extract (50 µg/ml and 100 µg/ml), HPPA (5 µg/ml and 10 µg/ml), or no-treatment control, in the presence or absence of an inflammatory stimulus (IL-1β), for a total of 8 treatment groups (*n* = 3/group). The data were obtained using the nCounter^Ⓡ^ Human v3 miRNA Expression Panel (NanoString Technologies, Seattle, WA, USA) (799 miRNAs, miRBase built version 21) as described in the supporting manuscript [1] and below. The raw miRNA data were deposited at the NCBI Gene Expression Omnibus (GEO) database under the data ID: GSE237078. This dataset provides the matrices of the normalized log2 transformed expression counts of the 799 miRNAs expressed in the Caco-2BBe1 cells in response to each treatment group (PAC, HPPA) stimulated with interleukin (IL)-1ß or not for 36 samples. These matrices can be used for further downstream analyses. Additional data have been compiled in three tables, described below.

Supplementary Table 1 lists the miRNAs found to be differentially expressed in response to each treatment (PAC, 50 µg/ml; HPPA, 5 µg/ml) vs control, in response to different concentrations of the same treatment (PAC, 50 µg/ml and 100 µg/ml; HPPA, 5 µg/ml and 10 µg/ml) and in response to IL-1β. Every comparison is presented in a separate tab. Supplementary Table 2 lists the putative gene targets of miRNAs that are listed in Supplementary Table 1. For clarity, each of the 15 tabs of Supplementary Table 2 aligns with the corresponding treatment group comparisons in Supplementary Table 1. Supplementary Table 3 lists the significantly enriched target pathways of the putative gene targets (*q <* 0.05) identified in supplementary Table 2. Each of the 15 tabs of Supplementary Table 3 aligns with the corresponding treatment group comparisons in Supplementary Table 1 and 2.

**Fig. 1:**
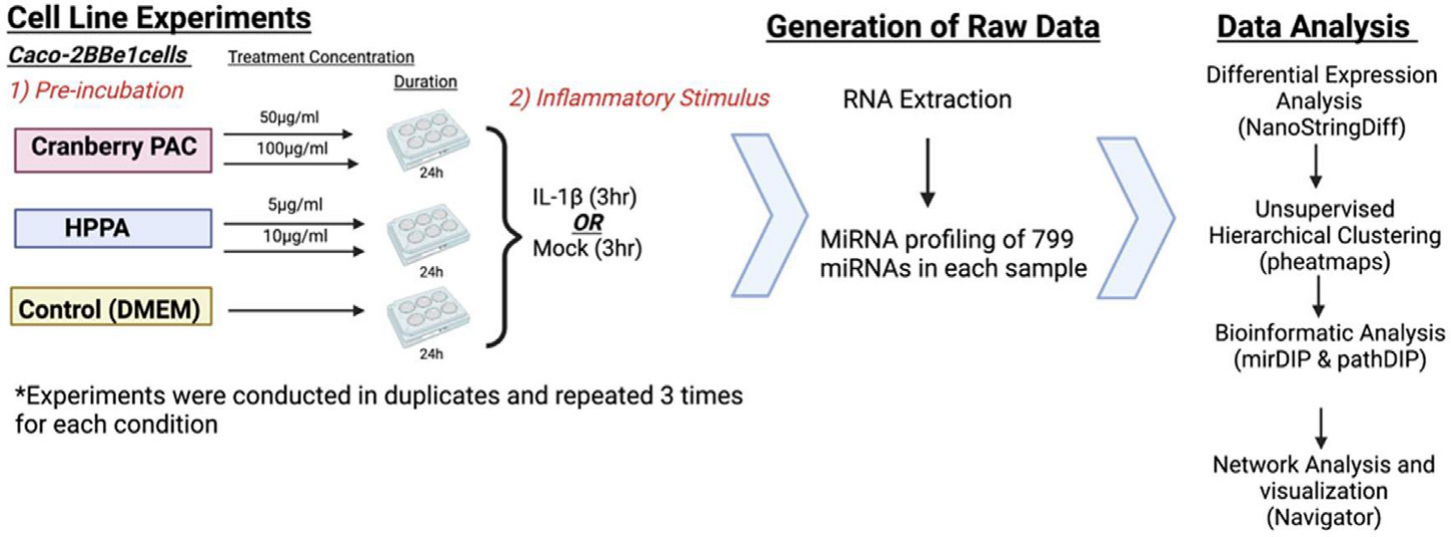
Experimental and analytical study design. Caco-2BBe1 cells were treated with two doses of PAC (50 µg/ml, 100 µg/ml), HPPA (5 µg/ml, 10 µg/ml), or with Dulbecco's Modified Eagle Medium (control) for 24 h, followed by IL-1β (1 ng/ml) to generate an inflammatory environment for three hours. Cells were then collected and used for total RNA extraction, and miRNA profiling. Details for the bioinformatics tools utilized are provided in the text. Image Created with BioRender.com.

**Fig. 2:**
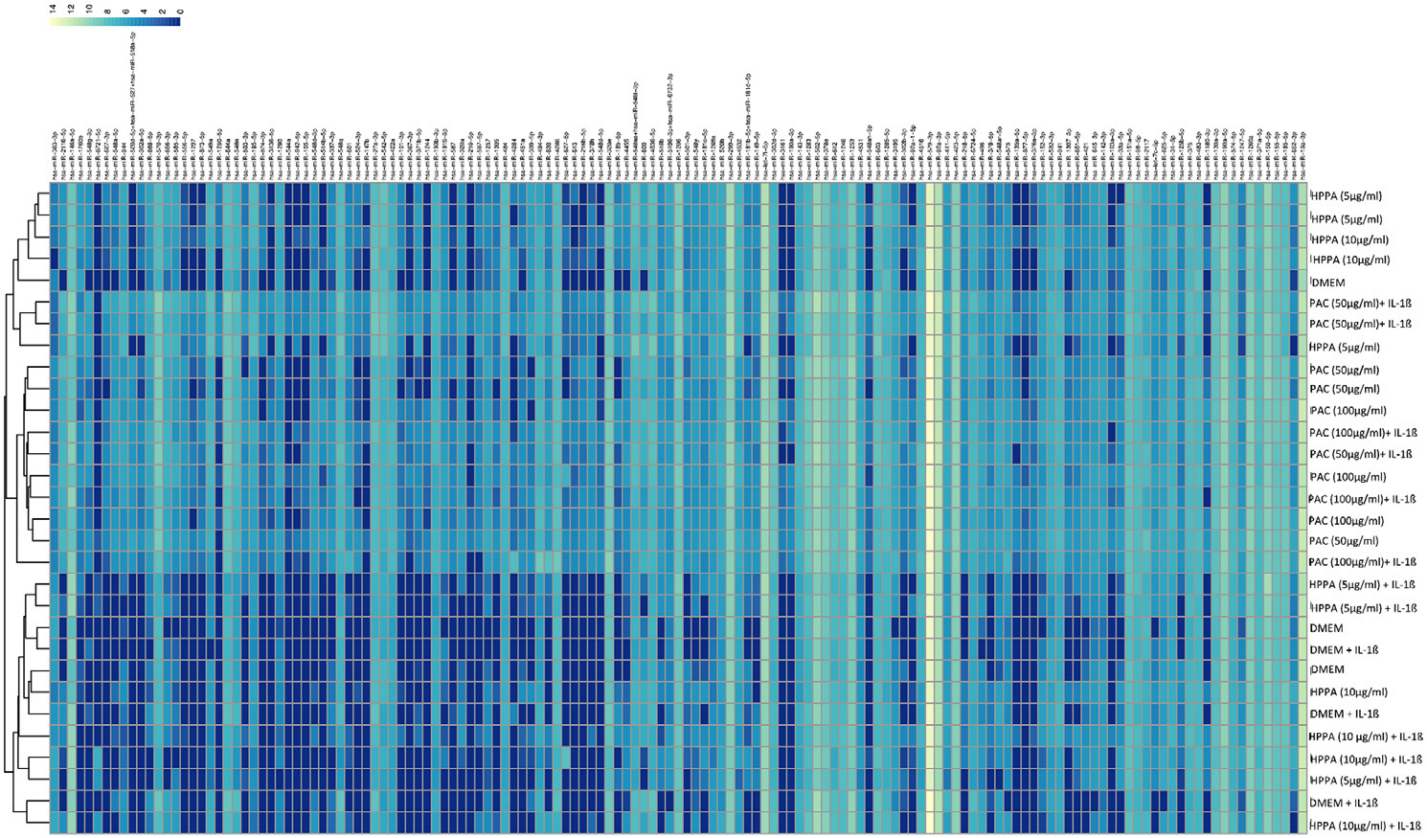
Heatmap of miRNAs affected by PAC and HPPA in both the inflammatory and non-inflammatory state. Hierarchical clustering analysis-based on the expression profiles of the significantly differentially expressed (*q <* 0.001) miRNAs in response to PAC, HPPA, DMEM at increasing concentrations in both the inflammatory (IL-1ß) and non-inflammatory state (*q <* 0.001), *n* = 3/group. Each row represents treatment conditions, each column represents miRNA expression. Colours represent expression levels: yellow, higher expression; blue, lower expression. N = 3/group.

**Fig. 3:**
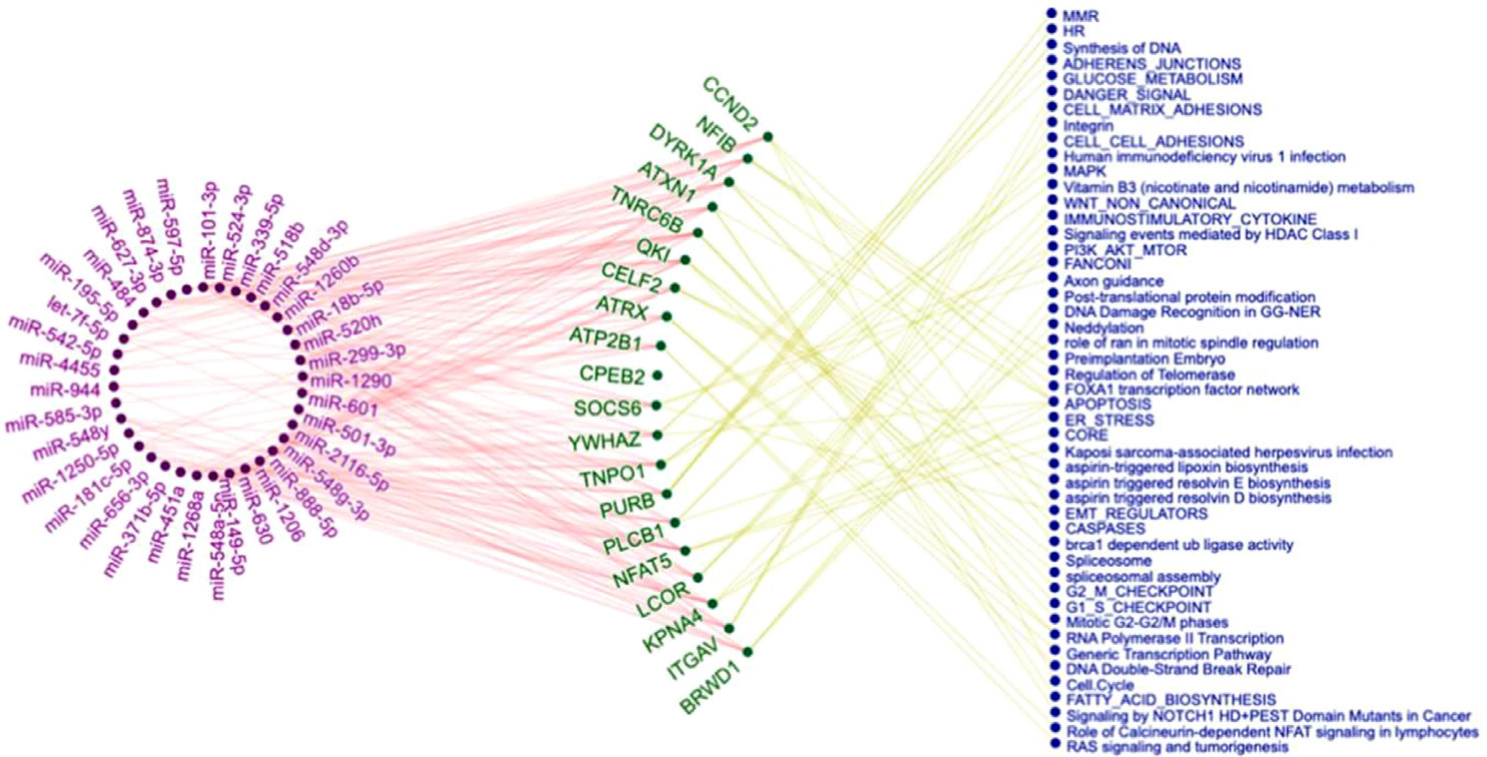
Network of PAC-responsive miRNA-gene target-pathway. The genes shown are the predicted (mirDIP) gene targets of miRNAs differentially expressed (*q <* 0.001) in response to increasing PAC concentration (50 µg/ml vs 100 µg/ml) in the non-inflammatory state. For readability, only genes targeted by 8 or more miRNAs, and their top 3 enriched pathways are shown. The full lists of gene targets and pathways are provided in Supplementary Tables 2 and 3. MiRNAs are depicted in purple, putative gene targets in green and pathways in blue.

**Fig. 4:**
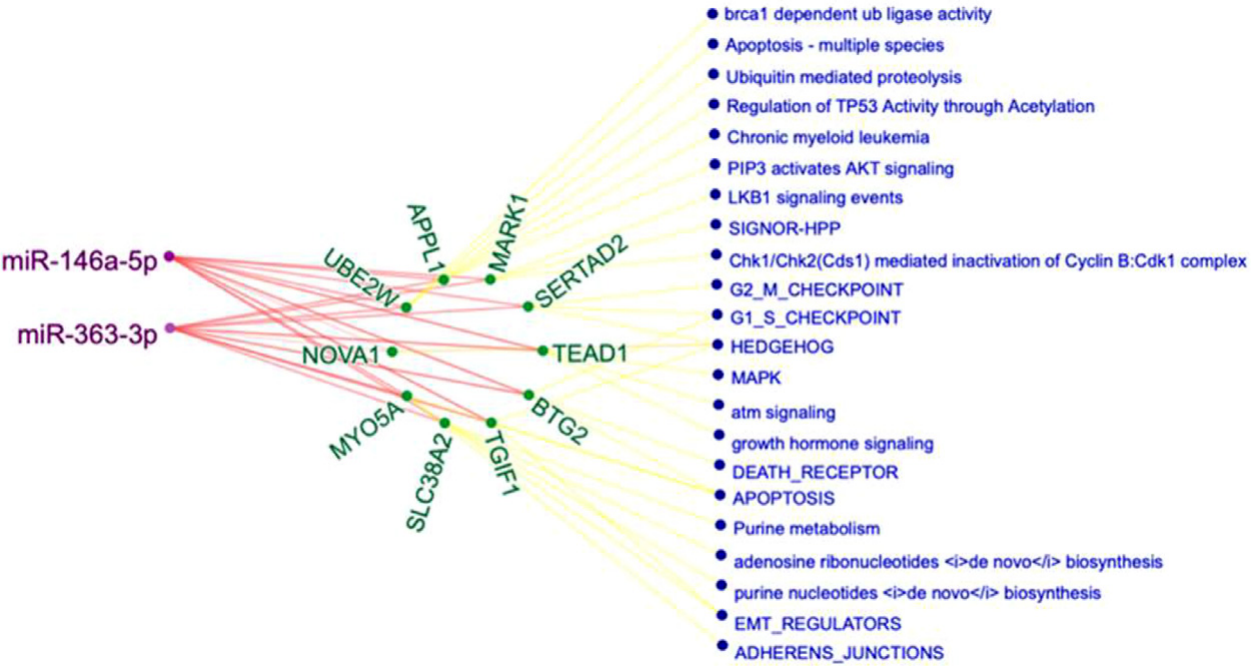
Network of HPPA-responsive miRNA-gene target-pathway. The genes shown are the predicted (mirDIP) gene targets of differentially expressed (*q <* 0.001) miRNAs in response to increasing HPPA concentrations (5 µg/ml vs 10 µg/ml) in the non-inflammatory state. For readability, only genes targeted by 2 or more miRNAs, and their top 3 target pathways were included. The full lists of gene targets and pathways are provided in Supplementary Tables 2 and 3. MiRNAs are depicted in purple, putative gene targets in green and pathways in blue.

Fig. 1 visualizes the study design and data generation plan. Fig. 2 is a heatmap created using correlation distances to show the unsupervised hierarchical clustering of the significantly differentially expressed miRNAs (*q <* 0.001) among treatments and concentrations listed in Supplementary Table 1. Fig. 3 visualizes the miRNA-gene target-pathway network for the significantly differentially expressed (*q <* 0.001) miRNAs identified in the treatment group comparison PAC 50 µg/ml vs PAC 100 µg/ml (no IL-1β) from Supplementary Table 1. Fig. 4 visualizes the miRNA-gene target-pathway network for the significantly differentially expressed (*q <* 0.001) miRNAs identified in the treatment group comparison HPPA 5 µg/ml vs HPPA 10 µg/ml (no IL-1β) from Supplementary Table 1.

## 3 Experimental Design, Materials and Methods

### 3.1 Cell line experiments

The procedures used for cell culture experiments have been described in detail in the supporting manuscript [1]. In short, fully differentiated Caco-2BBe1 cells (American Type Culture Collection, Manassas, VA, USA) were incubated with two concentrations of PAC (50 µg/ml or 100 µg/ml) (Ocean Spray Inc, Middleborough, MA, USA) or HPPA (5 µg/ml or 10 µg/ml) (Sigma-Aldrich, St. Louis, MO, USA, cat #11569-25MG), or with Dulbecco's Modified Eagle Medium (DMEM, mock; cat. #319-005-CL) for 24 h. The cells were then stimulated with IL-1ß (1 ng/ml) (Cell Signalling Technology, Danvers MA, USA, cat. #8900SF) or not (DMEM, mock) for three hours. Prior to the RNA extraction and miRNA profiling, the pellets were stored at -80 °C [1]. These experiments were performed in duplicate and repeated three times, producing 3 biological replicates for a total of 36 samples.

### 3.2 RNA extraction and miRNA profiling

The total RNA extraction and miRNA profiling procedures are provided in the supported manuscript [1]. To summarize, total RNA was extracted from the Caco-2BBe1 cells using the mirVana ™ miRNA Isolation Kit (Catalog No. AM1561, Ambion, Life Technologies, Waltham, MA, USA). 799 miRNAs were profiled using the nCounter Human v3 miRNA Expression Assay Kit (Catalog No. CSO-MIR3-12, NanoString Technologies, Seattle, WA, USA) (miRBase built version 21) at the Princess Margaret Genomics Centre, Toronto, ON. The processing and statistical analysis of the raw data were performed in R version 3.51 and R version 4.2.1 [2], using the R packages NanostringDiff, NanostringNorm, and Pheatmap [3], [4], [5]. The steps were as follows: The raw data of the 799 profiled miRNAs were loaded into R as a singular csv file. Contrast vectors were created to specify the comparisons made between each treatment group. The comparisons were ran using the r-script provided in the supporting manuscript and the results were saved to a csv file containing the number of differential expressed miRNAs. MiRNAs were considered differentially expressed at a q-value <0.001. Heatmaps were created to cluster and visualize the significantly differentially expressed miRNAs.

### 3.3 In silico gene target and pathway analysis

The putative gene targets of the significantly differentially expressed miRNAs were identified using the miRNA Data Integration Portal (mirDIP) [6]. For each treatment, the corresponding enriched pathways were identified using the Data Integration Portal (pathDIP) [7] and a Benjamini-Hochberg adjusted q-value < 0.05. NAViGaTOR [8] was used to create and visualize the networks linking the differentially expressed miRNAs with their gene targets and enriched pathways.

## CRediT authorship contribution statement

**Zoe Dimoff:** Conceptualization, Methodology, Data curation, Writing – original draft, Visualization, Investigation. **Zoe Lofft:** Conceptualization, Methodology, Data curation, Writing – original draft, Investigation. **Fred Liang:** Methodology, Data curation, Investigation, Writing – review & editing. **Siying Chen:** Methodology, Investigation, Writing – review & editing. **Paraskevi Massara:** Methodology, Writing – review & editing. **Diana Wu:** Methodology, Writing – review & editing. **Inke Paetau-Robinson:** Methodology, Resources, Writing – review & editing. **Christina Khoo:** Methodology, Resources, Writing – review & editing. **Amel Taibi:** Conceptualization, Supervision, Data curation, Writing – review & editing. **Elena M. Comelli:** Conceptualization, Supervision, Writing – review & editing, Project administration, Funding acquisition.

## Data Availability

miRNA expression data (Original data) (NCBI GEO).

## Appendix Supplementary materials

Image, application 1

Image, application 2

Image, application 3
